# Transcriptional Regulation of VEGFA by the Endoplasmic Reticulum Stress Transducer OASIS in ARPE-19 Cells

**DOI:** 10.1371/journal.pone.0055155

**Published:** 2013-01-30

**Authors:** Hidetaka Miyagi, Soshi Kanemoto, Atsushi Saito, Rie Asada, Hideo Iwamoto, Soutarou Izumi, Miori Kido, Fumi Gomi, Kohji Nishida, Yoshiaki Kiuchi, Kazunori Imaizumi

**Affiliations:** 1 Department of Biochemistry, Graduate School of Biomedical and Health Sciences, Hiroshima University, Hiroshima, Japan; 2 Department of Ophthalmology and Visual Science, Graduate School of Biomedical and Health Sciences, Hiroshima University, Hiroshima, Japan; 3 Department of Ophthalmology, Graduate School of Medicine, Osaka University, Osaka, Japan; UAE University, Faculty of Medicine & Health Sciences, United Arab Emirates

## Abstract

**Background:**

Vascular endothelial growth factor-A (VEGFA) is the main mediator of angiogenesis. Angiogenesis plays important roles not only in many physiological processes, but also in the pathophysiology of many diseases. VEGFA is one of the therapeutic targets of treatment for ocular diseases with neovascularization. Therefore, elucidation of the regulatory mechanisms for VEGFA expression is important for the development of pharmaceutical drugs. Recent studies have demonstrated that the unfolded protein response is involved in the transcriptional regulation of VEGFA. However, the precise regulation of VEGFA in the human retina is not fully understood.

**Principal Findings:**

When human retinal pigment epithelial cells, ARPE-19, were exposed to endoplasmic reticulum stressors, VEGFA mRNA was significantly upregulated. The unfolded protein response-related transcription factors XBP1, ATF4, ATF6, and OASIS were expressed in ARPE-19 cells. To determine which transcription factors preferentially contribute to the induction of VEGFA expression after endoplasmic reticulum stress, we carried out reporter assays using an approximately 6-kbp 5′-upstream region of the human VEGFA gene. Among these transcription factors, OASIS acted most effectively on the VEGFA promoter in ARPE-19 cells. Based on data obtained for certain deleted and mutated reporter constructs, we determined that OASIS promoted VEGFA expression by acting on a cyclic AMP-responsive element-like site located at around –500 bp relative to the VEGFA transcription start site. Furthermore, we confirmed that OASIS directly bound to the promoter region containing this site by chromatin immunoprecipitation assays.

**Conclusions and Significance:**

We have demonstrated a novel regulatory mechanism for VEGFA transcription by OASIS in human retinal pigment epithelial cells. Chemical compounds that regulate the binding of OASIS to the promoter region of the VEGFA gene may have potential as therapeutic agents for ocular diseases with neovascularization.

## Introduction

The endoplasmic reticulum (ER) is an organelle responsible for the synthesis, folding, and post-translational modifications of secretory and transmembrane proteins. Various cellular stresses, including oxidative stress, ischemic insults, and expression of mutated genes, lead to the accumulation of unfolded or misfolded proteins in the ER lumen, and to impairment of ER functions. These states are termed ER stress [1], [2]. Eukaryotic cells have a protective system to cope with ER stress, which is composed of translational attenuation, upregulation of ER chaperones to facilitate protein folding, and promotion of the degradation of unfolded proteins (ER-associated degradation; ERAD). This system is called the unfolded protein response (UPR) [3]–[5]. Mammalian cells have three canonical ER stress transducers; PKR-like endoplasmic reticulum kinase (PERK) [6], inositol-requiring enzyme 1 (IRE1) [7], [8], and activating transcription factor 6 (ATF6) [9], [10]. These ER stress transducers are transmembrane proteins that localize to the ER membrane and monitor the status of the ER lumen. When cells are exposed to ER stress, PERK phosphorylates eukaryotic initiation factor 2 α (eIF2α), a translational complex subunit, followed by translational attenuation. On the other hand, and paradoxically, phosphorylation of eIF2α also upregulates the expression of ATF4 [4]. ATF4 transactivates the expression of both a pro-apoptotic protein, CHOP, and pro-survival proteins, such as ER chaperones and anti-oxidative stress proteins [11]. IRE1 processes unspliced forms of X-box-binding protein-1 (XBP1) mRNA to generate spliced forms of the mRNA [7], [8], [12]–[14]. XBP1 proteins derived from the spliced forms of XBP1 mRNA induce the expression of ER-resident chaperones and ERAD-related molecules [15], [16]. ATF6 is cleaved at its transmembrane region by site-1 and site-2 proteases in response to ER stress [10], [17]. The cleaved ATF6 N-terminus translocates into the nucleus and induces the expression of ER-resident chaperones to facilitate protein folding.

Recently, novel ER stress transducers that are structurally included in the CREB/ATF family (OASIS family) were identified. These are OASIS, BBF2H7, CREBH, CREB4, and Luman, which share a region of high sequence similarity with ATF6 [18], [19]. The features of these molecules are cell- or tissue-specific expression patterns. For example, OASIS is preferentially expressed in osteoblasts and astrocytes [20]–[25], and is involved in its terminal differentiation [26]–[29]. Thus, new branches of the UPR composed of these ER stress transducers could provide important signals for regulating cell differentiation and maturation or the maintenance of basal cellular homeostasis [18], [19], [30], [31].

Angiogenesis consists of the sprouting, migration, and remodeling of existing blood vessels [32] and plays important roles in various normal physiological processes. However, angiogenesis also occurs in several pathological conditions and causes many diseases. Hypoxic or chronic inflammatory conditions provoke undesired angiogenesis. In the ophthalmologic field, this unwanted angiogenesis leads to severe ocular diseases, such as diabetic retinopathy, neovascular glaucoma, and age-related macular degeneration. Therefore, prevention of angiogenesis in the retina and choroid is important for treating these diseases. Angiogenesis is regulated by a fine balance between factors that stimulate and inhibit the formation of new blood vessels [33], [34]. Vascular endothelial growth factor-A (VEGFA) is the major and best-studied proangiogenic factor. This molecule is a homodimeric heparin-binding glycoprotein, and has several isoforms produced from the VEGFA gene by alternative splicing. The VEGFA isoforms show various expression patterns and contrasting characteristics [35]. All of the VEGFA isoforms are synthesized and processed in the ER and transported to the cell membrane through the secretory pathway [36],[37]. Several recent studies have demonstrated that the UPR is involved in VEGFA transcription [38], [39]. Some of the UPR components, XBP1 and ATF4, play roles in the promotion of VEGFA transcription in response to ER stress. However, it has not been clarified whether the other UPR components, including OASIS family members, regulate or modulate VEGFA transcription under ER stress conditions. Here, we report that the novel ER stress transducer OASIS acts on the promoter region of the VEGFA gene and facilitates its transcription in human retinal pigment epithelial cells.

## Results

### VEGFA Expression is Induced by ER Stress in ARPE-19 Cells

**Figure 1:**
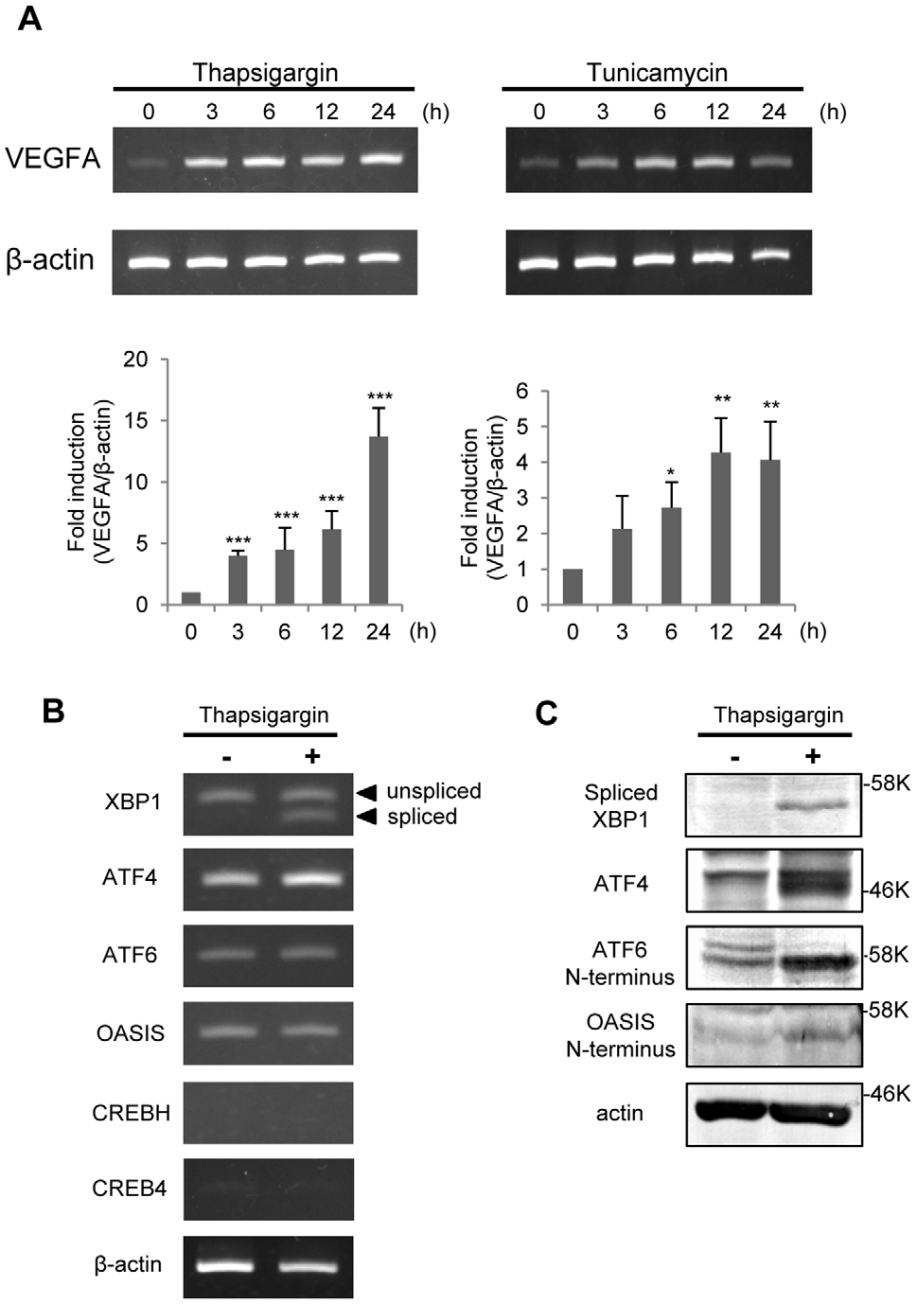
VEGFA mRNA is upregulated by ER stressors. (A) RT-PCR analysis of VEGFA and β-actin in ARPE-19 cells treated with ER stressors (1 µM thapsigargin or 3 µg/ml tunicamycin) for 3, 6, 12, and 24 h. The bottom panels show the results of real-time RT-PCR. Data are means ± SD (n = 3). *p<0.05, **p<0.01, ***p<0.001, by Student’s *t*-test. (B) RT-PCR analyses of UPR-related transcription factors in ARPE-19 cells under normal condition and ER stress with 1 µM thapsigargin for 6 h. Unspliced; unspliced forms of XBP1 mRNA, spliced; spliced forms of XBP1 mRNA. (C) Western blot analyses of XBP1, ATF4, ATF6, and OASIS in ARPE-19 cells under ER stress with 1 µM thapsigargin for 12 h. Activated forms of these four molecules were upregulated under ER stress conditions.

To check the response of VEGFA expression to ER stress in ARPE-19 cells, we treated the cells with ER stress inducers, 1 µM thapsigargin or 3 µg/ml tunicamycin, for 3, 6, 12, and 24 h. Total RNA was isolated from the cells, and subjected to RT-PCR analysis (Figure 1A). Treatment with thapsigargin and tunicamycin significantly increased the VEGFA mRNA expression levels by 4–13-fold compared with non-treated control cells, indicating that VEGFA expression is regulated by UPR signaling. Next, we examined which UPR-related transcription factors are involved in the expression of VEGFA mRNA in ARPE-19 cells. We checked the expression of several transcription factors, XBP1 in the IRE1 pathway, ATF4 in the PERK pathway, ATF6, and OASIS family members including OASIS, CREBH, and CREB4, under normal and ER stress conditions. In both conditions, XBP1, ATF4, ATF6, and OASIS were expressed in ARPE-19 cells, while CREBH and CREB4 were not (Figure 1B). Western blot analyses showed that the former four factors were activated under ER stress conditions, comprising upregulation of XBP1 proteins derived from the spliced mRNA forms and translated ATF4, and increases in the N-terminal fragments of ATF6 and OASIS (Figure 1C). These findings suggest that specific UPR components are expressed and activated in ARPE-19 cells under ER stress and could affect the expression of VEGFA mRNA.

### Structure of the Human VEGFA Promoter and Effects of OASIS on its Activities

**Figure 2:**
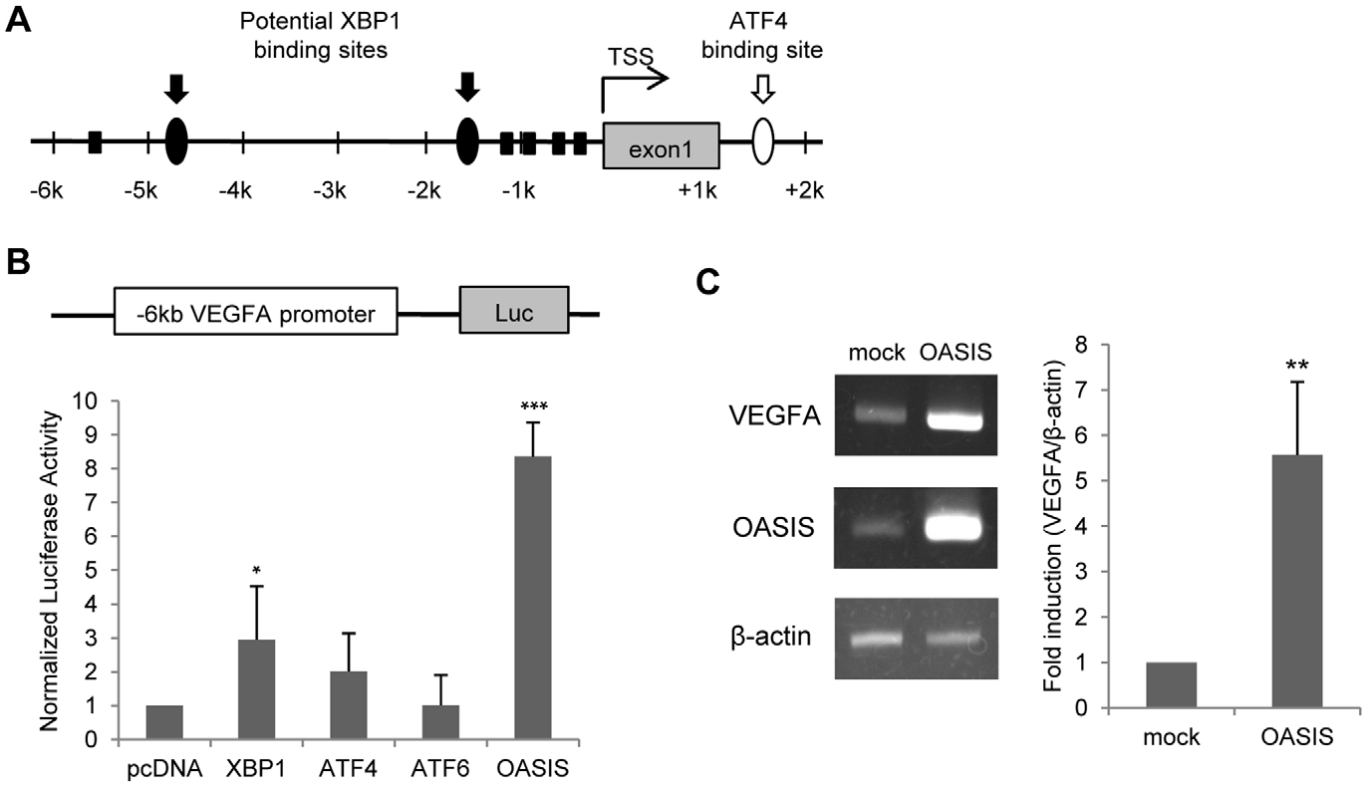
OASIS promotes the transcription of VEGFA. (A) Schematic diagram of the human VEGFA promoter region. The first intron of the human VEGFA gene contains an ATF4-binding site (○), and that the 6-kbp 5′-upstream promoter has two potential binding sites for XBP1 (•) and CRE-like sites (▪). TSS: transcription start site. (B) Reporter assays using ARPE-19 cells. A reporter vector derived from the 6-kbp 5′-upstream region of the human VEGFA gene and expression vectors for XBP1, ATF4, ATF6, or OASIS were co-transfected. At 48 h after the transfection, luciferase activities were measured. Data are means ± SD (n = 4). *p<0.05, ***p<0.001, by Student’s *t*-test. (C) RT-PCR analysis of VEGFA in ARPE-19 cells infected with an adenovirus vector carrying OASIS. The right panel shows the results of real-time RT-PCR. The VEGFA mRNA expression level is increased by 5.5-fold after the transfection of OASIS. Data are means ± SD (n = 3). **p<0.01, by Student’s *t*-test.

To analyze the regulation of VEGFA expression in ARPE-19 cells, we carried out reporter assays using VEGFA promoter regions. Two potential XBP1-binding sites within the 6-kbp 5′-upstream region and one ATF4-binding site in the first intron are known to be present around the transcription start site of the human VEGFA gene [39]. In addition, we found five cAMP-responsive element (CRE)-like sites in the 6-kbp 5′-upstream region of the VEGFA gene (Figure 2A), which are elements that OASIS can bind to [20], [26]. Therefore, we focused on the transcriptional regulation of the 5′-upstream region of the VEGFA gene by OASIS. A 6-kbp 5′-upstream sequence (–5868 to +313 bp) from the human VEGFA transcription start site was cloned into the pGL3-basic reporter plasmid (pGL3-hVEGFA promoter 6 kbp). This reporter plasmid and each UPR-related transcription factor expression vector were co-transfected into ARPE-19 cells, and the luciferase activities were measured. Introduction of the OASIS and XBP1 expression vectors significantly increased the luciferase activities, and OASIS was the most effective among the UPR-related transcription factors (Figure 2B). These findings indicate that OASIS may be the most important factor for the transcription of human VEGFA in ARPE-19 cells.

To confirm that OASIS induces the expression of endogenous VEGFA in ARPE-19 cells, we examined the VEGFA expression levels in ARPE-19 cells infected with an adenovirus expressing the N-terminus of OASIS (Figure 2C). The VEGFA mRNA levels were significantly elevated in the OASIS-infected cells. Taken together, these findings indicate that OASIS acts on the 6-kbp promoter region of the VEGFA gene and promotes the expression of VEGFA mRNA in ARPE-19 cells.

### OASIS Modulates Human VEGFA Promoter Activities via a CRE-like Site

**Figure 3:**
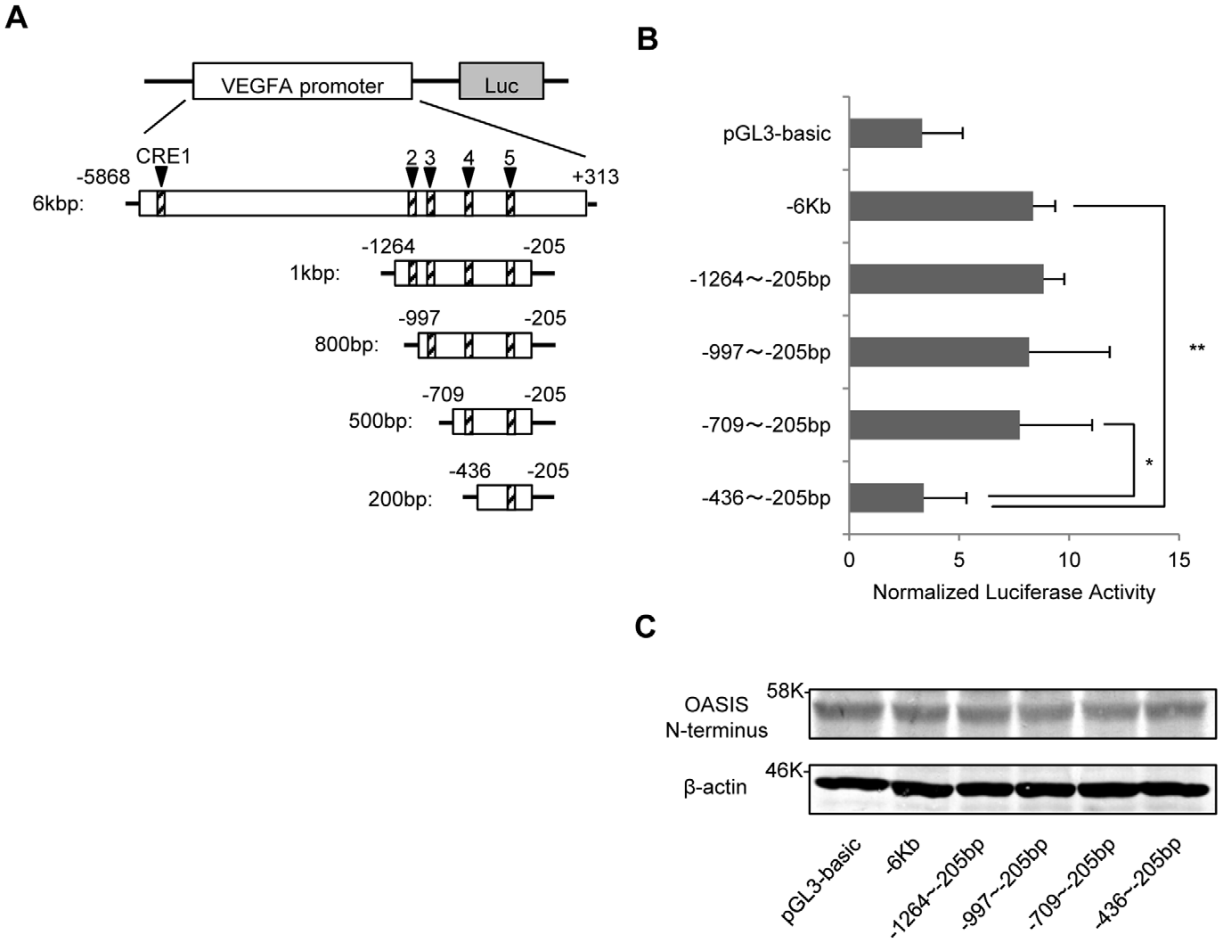
OASIS modulates VEGFA promoter activities via the region between –709 and –437 bp. (A) Schematic diagrams of the deleted reporter constructs from the 6-kbp 5′-upstream promoter of the human VEGFA gene. Five putative CRE-like sites (containing an ACGT core) exist in the 6-kbp VEGFA promoter region. (B) Reporter assays using ARPE-19 cells. Each deletion reporter vector and the OASIS N-terminus expression vector were co-transfected. Reporter assays were performed at 48 h after the transfection. Note that reporter activities significantly decreased in cells transfected with the 200-bp construct, suggesting that OASIS acts on a site in the region between –709 and –437 bp of the VEGFA promoter. Data are means ± SD (n = 6). *p<0.05, **p<0.01, by Student’s *t*-test. (C) Western blot analysis shows the FLAG-tagged OASIS N-terminus was expressed at equal levels in each sample.

Among the five CRE-like sites in the 6-kbp promoter of the VEGFA gene, we tried to determine the sites that OASIS specifically acted on. First, we constructed truncated reporter genes that were deleted from the original 6-kbp human VEGFA promoter and contained different numbers of CRE-like sites (Figure 3A). Although the luciferase activities in ARPE-19 cells transfected with the 500-bp (–709 to –205 bp) reporter construct were equal to those in cells transfected with the full-length 6-kbp construct, those in cells transfected with the 200-bp (–436 to –205 bp) construct were dramatically reduced (Figure 3B). This indicates that OASIS acts on the sequence from –709 to –437 bp in the VEGFA promoter to facilitate the reporter activities.

**Figure 4:**
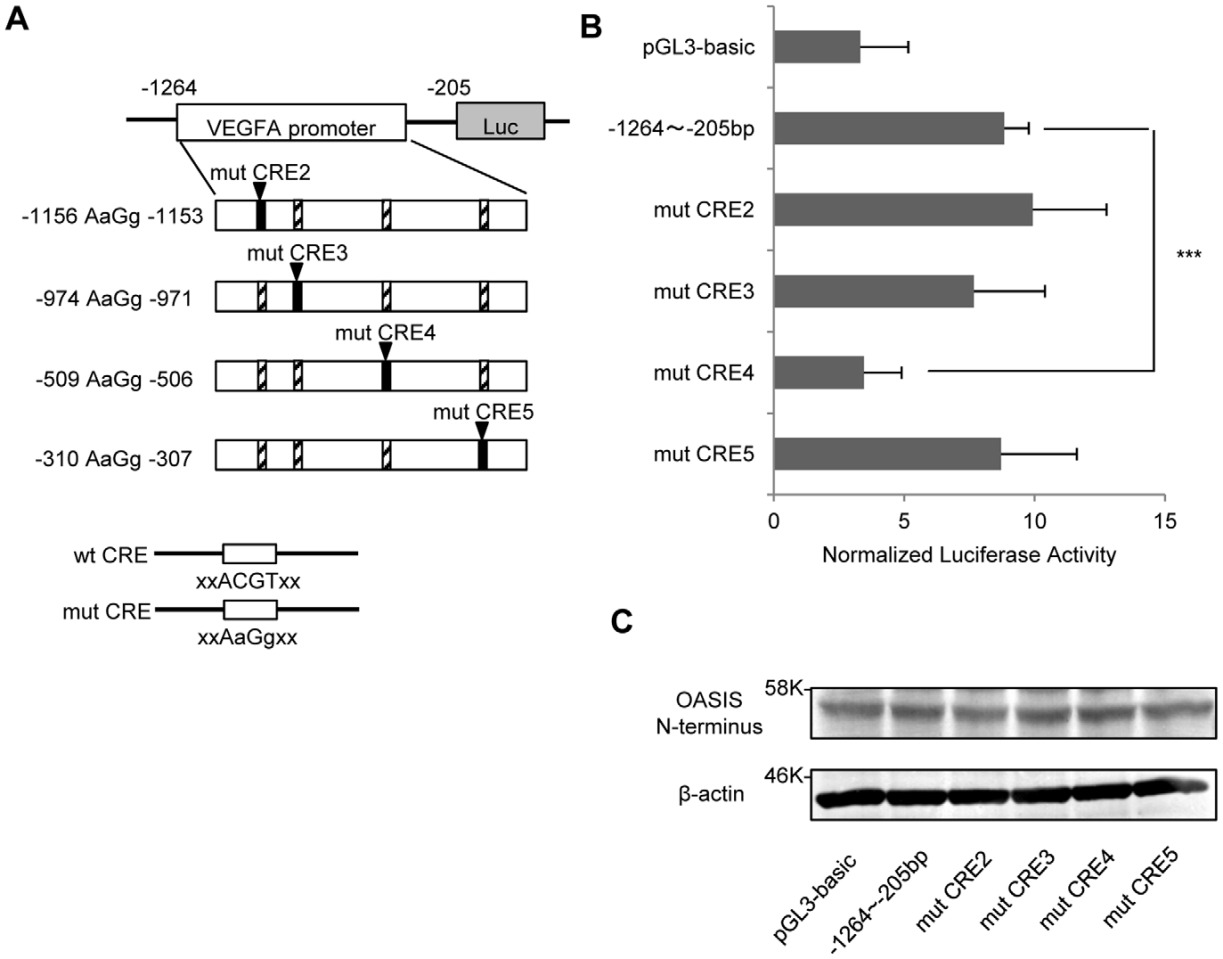
OASIS acts on the CRE-like site at –509 to –506 bp in the VEGFA promoter. (A) The top panel shows schematic diagrams of the mutated reporter constructs. The bottom panel shows schematic representations of the wild-type CRE-like site (containing an ACGT core) and the mutated CRE-like sites (containing an AaGg core). (B) Reporter assays using ARPE-19 cells. Each mutated reporter vector and the OASIS N-terminus expression vector were co-transfected. Reporter assays were performed at 48 h after the transfection. Note that reporter activities significantly decreased in cells transfected with the mutated CRE-like site 4 construct. Data are means ± SD (n = 4). ***p<0.001, by Student’s *t*-test. (C) Western blot analysis shows the FLAG-tagged OASIS N-terminus was expressed at equal levels in each sample.

Next, to identify the CRE-like sites that OASIS acts upon, we generated mutated reporter constructs that were exchanged from the ACGT core sequence to the AaGg sequence in each CRE-like site (Figure 4A). The reporter activities in cells transfected with the mutated CRE-like site 4 (–509 AaGg –506) construct were significantly reduced, while those in cells transfected with the other mutated constructs were promoted by OASIS (Figure 4B). These findings indicate that OASIS specifically acts on CRE-like site 4 between –509 and –506 bp in the human VEGFA promoter to activate its transcription.

### OASIS Directly Binds to the Promoter Region in the Human VEGFA Gene

**Figure 5:**
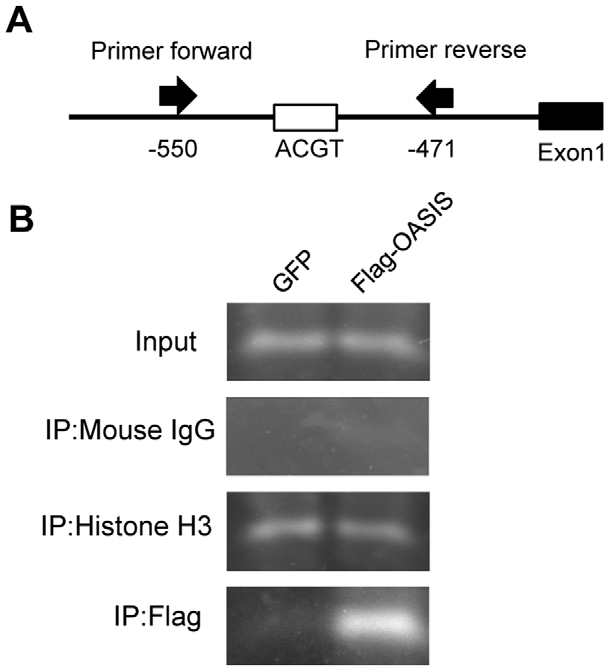
OASIS directly binds to the promoter region in the human VEGFA gene. (A) Schematic representation of the VEGFA promoter and the annealing sites of the primer set used in the ChIP assays. (B) PCR amplification of the VEGFA promoter region including the CRE-like site 4. ARPE-19 cells were transfected with a vector expressing the FLAG-tagged OASIS N-terminus. A GFP expression vector was used as a control. Immunoprecipitation was performed with anti-histone H3, anti-mouse IgG, or anti-FLAG antibodies, followed by the PCR using the specific primer sets.

To confirm that OASIS directly binds to the promoter region including CRE-like site 4, we performed chromatin immunoprecipitation (ChIP) assays. ARPE-19 cells were transfected with expression vectors for FLAG-tagged OASIS N-terminus or green fluorescent protein (GFP), followed by immunoprecipitation with anti-histone H3, anti-mouse IgG, or anti-FLAG antibodies. The region of –550 to –471 bp in the human VEGFA promoter containing CRE-like site 4 was then amplified from the precipitated DNA using a specific primer set (Figure 5A). Specific bands were detected in the anti-histone H3 antibody immunoprecipitates of lysates from both GFP- and FLAG-OASIS-transfected cells, but not in the anti-mouse IgG antibody immunoprecipitates. When the anti-FLAG antibody was used for immunoprecipitation, the specific amplified band was observed in cells transfected with FLAG-OASIS expression vectors (Figure 5B). These findings suggest that OASIS directly binds to the promoter region including CRE-like site 4 in the human VEGFA gene.

## Discussion

Previous studies showed that UPR signaling affects the transcription of VEGFA mRNA [38], [39]. It was suggested that XBP1 facilitates the promoter activities by acting on the 5′-upstream region of the VEGFA gene and that ATF4 promotes these activities by acting on the first intron. In addition, in IRE1α^−/−^ or XBP1^−/−^ mouse embryonic fibroblasts (MEFs), the VEGFA expression levels were significantly reduced under ER stress conditions. These levels were also decreased in ATF4^−/−^ MEFs. Interestingly, however, the VEGFA induction in these cells was partially decreased and did not completely disappear [38], [39]. We also confirmed that the decreases in VEGFA expression were small in both IRE1α/β^−/−^ and PERK^−/−^ MEFs (data not shown). These observations allowed us to propose the possibility that other UPR-related transcription factors are also involved in the transcriptional regulation of VEGFA.

In the present study, we have demonstrated that OASIS promotes the expression of VEGFA by directly binding to its promoter region in ARPE-19 cells. The reasons for this conclusion are as follows: 1) OASIS was expressed in ARPE-19 cells and cleaved at the membrane region in response to ER stress; 2) OASIS facilitated the expression of a reporter gene carrying the 6-kbp 5′-upstream promoter region in the VEGFA gene; 3) OASIS acted on a specific region, –709 to –437 bp, of the 5′-upstream promoter region; and 4) OASIS directly bound to the promoter region including CRE-like site 4, –509 to –506 bp. XBP1 and ATF4 belong to the CREB/ATF family as well as OASIS [18], [19]. It is well known that CREB/ATF family transcription factors form heterodimers between the individual molecules via their bZIP domains and promote the transcription of target genes [40]. Therefore, it is possible that OASIS spatiotemporally regulates the expression of VEGFA by forming heterodimers with XBP1 or ATF4. However, to clarify the detailed regulation of VEGFA expression under ER stress conditions, further studies including regulated complex formation of OASIS and XBP1 or ATF4 are needed.

VEGFA is one of the proangiogenic factors, and is involved in the pathophysiology of some ocular diseases with neovascularization. In fact, treatment of age-related macular degeneration patients with anti-VEGF humanized monoclonal antibodies successfully delays the progression of its pathology [41], [42]. Furthermore, it is generally accepted that anti-VEGF antibodies or triamcinolone acetonide, which suppresses VEGF levels [43], are effective treatment for diabetic retinopathy. Therefore, VEGFA is one of the best targets for the development of therapeutic strategies for these diseases. On the other hand, ER stress and UPR signaling are known to be related to the onset or progression of many ocular diseases, such as retinitis pigmentosa caused by mutated rhodopsin [44], primary open angle glaucoma [45], diabetic retinopathy [46], [47], and age-related macular degeneration [48]. Thus, modulators of ER stress or the UPR, including the OASIS signaling pathway, could be potent candidates for therapeutic strategies targeting ocular diseases with neovascularization. However, for the development of new therapeutic medicines for these diseases, it is essential to clarify the *in vivo* regulation of the UPR in patients.

## Materials and Methods

### Cell Culture, ER Stress Induction, and Virus Infection

ARPE-19 cells (derived from retinal pigment epithelium, an immortal non-transformed cell line from a human donor) were obtained from American Type Culture Collection (ATCC) and cultured in Dulbecco’s modified Eagle’s medium/F12 human amniotic membrane nutrient mixture (Gibco, Invitrogen) supplemented with 10% fetal bovine serum. The cultures were maintained in a humidified incubator at 37°C in an atmosphere containing 5% CO_2_. Thapsigargin (Sigma-Aldrich) and tunicamycin (Sigma-Aldrich) were dissolved in dimethylsulfoxide (DMSO; Sigma-Aldrich) to produce stock solutions. Cells were treated with 1 µM thapsigargin or 3 µg/ml tunicamycin for specified periods of time. To evaluate the effects of DMSO, medium containing only DMSO (1∶1000 of total volume) was also examined in each experiment. For adenovirus generation, a recombinant adenovirus carrying OASIS was constructed by homologous recombination between the expression cosmid cassette and the parental virus genome in HEK293 cells, as described previously [49], [50]. Cells were infected with the adenovirus at 48 h before analysis.

### RNA Isolation and RT-PCR

Total RNA was isolated from ARPE-19 cells using Isogen (Wako) according to the manufacturer’s protocol. First-strand cDNA was synthesized in 20 µl of reaction volume using a random primer (Takara) and Moloney murine leukemia virus reverse transcriptase (Invitrogen). PCR was performed using specific primer sets in a total volume of 30 µl containing 0.8 µM of each primer, 0.2 mM dNTPs, 3 U of Taq polymerase, and 10× PCR buffer (Agilent). The primer sequences are summarized in Table S1. The PCR products were resolved by electrophoresis in a 4.8% acrylamide gel.

Real-time PCR analyses were performed for 1-µl aliquots of the prepared cDNA samples using KAPA SYBR FAST qPCR Master Mix (Kapa Biosystems) and primers in a LightCycler 480 System II (Roche). The primer sequences are indicated in Table S1. The expression of each PCR product was quantified relative to the corresponding β-actin expression.

### Western Blotting

Proteins were extracted from ARPE-19 cells lysed with cell extraction buffer comprising 2.5 mM methionine, 33.3 mM Tris-acetate pH 8.5, 5 mM EDTA, 0.3% SDS, 1.5% Triton X-100, and protease inhibitor cocktail (MBL). The lysates were incubated on ice for 30 min. After centrifugation at 15,000 *g* for 10 min, the soluble protein concentrations were equalized using BCA protein assay reagents (Pierce). The following antibodies and dilutions were used: anti-actin (Millipore); anti-FLAG M5 (Sigma-Aldrich); anti-XBP1 (Santa Cruz Biotechnology); anti-ATF4 (Santa Cruz Biotechnology); and anti-ATF6 (Santa Cruz Biotechnology). The anti-OASIS monoclonal antibody was generated previously [26]. The density of each band was quantified using Photoshop Elements 2.0 (Adobe Systems).

### Reporter Plasmids and Luciferase Assay

The 6-kbp human VEGFA promoter (–5868 to +313 bp) was inserted into the pGL3-basic vector (Promega), and designated pGL3-hVEGFA promoter 6 kbp. All plasmids for VEGFA promoter deletion constructs and mutants were generated by a PCR-based approach using pGL3-hVEGFA promoter (6 kbp) as a template. ARPE-19 cells plated on 24-well plates were transfected with a reporter plasmid (0.1 µg) carrying the firefly luciferase gene and the reference plasmid pRL-SV40 (0.01 µg) carrying the *Renilla* luciferase gene under the control of the SV40 enhancer and promoter (Promega) together with 0.1 µg of each plasmid expressing an effector protein (mock pcDNA, FLAG-tagged OASIS N-terminus, XBP1 derived from spliced mRNA, ATF4, and ATF6 N-terminus) using Lipofectamine 2000 (Invitrogen). After 48 h, the cells were lysed in 100 µl of 5× Passive Lysis Buffer (Promega). The firefly luciferase and *Renilla* luciferase activities were measured in 10-µl aliquots of cell lysates using a Dual-Luciferase Reporter Assay System and Luminometer (Promega). The relative activity was defined as the ratio of firefly luciferase activity to *Renilla* luciferase activity. Each sample was normalized by the value produced in mock wells. The assays were performed independently more than four times.

### ChIP Assay

ARPE-19 cells were grown to 80% confluence in 10-cm dishes under normal cell culture conditions and then transfected with each expression plasmid (GFP or FLAG-tagged OASIS N-terminus) using an electroporation system (CUY21Vitro-EX; BEX) according to the manufacturer’s protocol. Protein–DNA crosslinking was initiated by directly adding formaldehyde to the culture medium at a final concentration of 1% and cells were incubated for 15 min. To harvest the cells, the plates were rinsed with cold PBS containing protease inhibitors and scraped. Chromatin was prepared using a ChIP Assay Kit (Upstate Biotechnology) according to the manufacturer’s protocol of 30 s × 30 strokes of sonication pulses, which yielded chromatin fragments with apparent sizes of 100–500 bp. An aliquot of each sample representing 5% of the total volume was removed for use as the input fraction and processed with the eluted immunoprecipitates beginning at the crosslink reversal step. Equal amounts of chromatin from each sample were incubated overnight at 4°C with 1 µl of anti-FLAG M2 (Sigma-Aldrich), anti-mouse IgG (Sigma-Aldrich) or anti-histone H3 (Santa Cruz Biotechnology) antibodies. Formaldehyde-induced cross-linking was reversed (>6 h at 65°C) and the DNA was purified by phenol–chloroform extraction and ethanol precipitation. The purified DNAs from the input and immunoprecipitated samples were subjected to 35 cycles of PCR. The PCR products were electrophoresed in 4.8% polyacrylamide gels and visualized by ethidium bromide staining. The primers used for the human VEGFA promoter were: 5′-AAGCTGGGTGAATGGAGCGA-3′ (forward) and 5′-CACACGCACACACTCACTCA-3′ (reverse), yielding an 80-bp product.

## Supporting Information

Table S1 **RT-PCR primer sequences.** The following table indicates sets of primers used for RT-PCR.
(DOC)

## References

[1] Rutkowski DT, Kaufman RJ (2004) A trip to the ER. Coping with stress. Trends Cell Biol 14: 20–28. 14729177 10.1016/j.tcb.2003.11.001

[2] Zhang K, Kaufman RJ (2008) From endoplasmic reticulum stress to the inflammatory response. Nature 454: 455–462. 18650916 10.1038/nature07203 PMC2727659

[3] Schröder M, Kaufman RJ (2005) ER stress and the unfolded protein response. Mutat Res 569: 29–63. 15603751 10.1016/j.mrfmmm.2004.06.056

[4] Ron D (2002) Translational control in the endoplasmic reticulum stress response. J Clin Invest 110: 1383–1388. 12438433 10.1172/JCI16784 PMC151821

[5] Kaufman RJ (2002) Orchestrating the unfolded protein response in health and disease. J Clin Invest 110: 1389–1398. 12438434 10.1172/JCI16886 PMC151822

[6] Harding HP, Zhang Y, Ron D (1999) Protein translation and folding are coupled by an endoplasmic-reticulum-resident kinase. Nature 397: 271–274. 9930704 10.1038/16729

[7] Tirasophon W, Welihinda AA, Kaufman RJ (1998) A stress response pathway from the endoplasmic reticulum to the nucleus requires a novel bifunctional protein kinase/endoribonuclease (Ire1p) in mammalian cells. Genes Dev 12: 1812–1824. 9637683 10.1101/gad.12.12.1812 PMC316900

[8] Calfon M, Zeng H, Urano F, Till JH, Hubbard SR, et al (2002) IRE1 couples endoplasmic reticulum load to secretory capacity by processing the XBP-1 mRNA. Nature 415: 92–96. 11780124 10.1038/415092a

[9] Yoshida H, Okada T, Haze K, Yanagi H, Yura T, et al (2000) ATF6 activated by proteolysis binds in the presence of NF-Y (CBF) directly to the cis-acting element responsible for the mammalian unfolded protein response. Mol Cell Biol 20: 6755–6767. 10958673 10.1128/mcb.20.18.6755-6767.2000 PMC86199

[10] Shen J, Chen X, Hendershot L, Prywes R (2002) ER stress regulation of ATF6 localization by dissociation of BiP/GRP78 binding and unmasking of Golgi localization signals. Dev Cell 3: 99–111. 12110171 10.1016/s1534-5807(02)00203-4

[11] Harding HP, Zhang Y, Zeng H, Novoa I, Lu PD, et al (2003) An integrated stress response regulates amino acid metabolism and resistance to oxidative stress. Mol Cell 11: 619–633. 12667446 10.1016/s1097-2765(03)00105-9

[12] Wang XZ, Harding HP, Zhang Y, Jolicoeur EM, Kuroda M, et al (1998) Cloning of mammalian Ire1 reveals diversity in the ER stress response. EMBO J 17: 5708–5717. 9755171 10.1093/emboj/17.19.5708 PMC1170899

[13] Yoshida H, Matsui T, Yamamoto A, Okada T, Mori K (2001) XBP1 mRNA is induced by ATF6 and spliced by IRE1 in response to ER stress to produce a highly active transcription factor. Cell 107: 881–891. 11779464 10.1016/s0092-8674(01)00611-0

[14] Yoshida H, Matsui T, Hosokawa N, Kaufman RJ, Nagata K, et al (2003) A time-dependent phase shift in the mammalian unfolded protein response. Dev Cell 4: 265–271. 12586069 10.1016/s1534-5807(03)00022-4

[15] Lee AH, Iwakoshi NN, Glimcher LH (2003) XBP-1 regulates a subset of endoplasmic reticulum resident chaperone genes in the unfolded protein response. Mol Cell Biol 23: 7448–7459. 14559994 10.1128/MCB.23.21.7448-7459.2003 PMC207643

[16] Shaffer AL, Shapiro-Shelef M, Iwakoshi NN, Lee AH, Qian SB, et al (2004) XBP1, downstream of Blimp-1, expands the secretory apparatus and other organelles, and increases protein synthesis in plasma cell differentiation. Immunity 21: 81–93. 15345222 10.1016/j.immuni.2004.06.010

[17] Ye J, Rawson RB, Komuro R, Chen X, Davé UP, et al (2000) ER stress induces cleavage of membrane-bound ATF6 by the same proteases that process SREBPs. Mol Cell 6: 1355–1364. 11163209 10.1016/s1097-2765(00)00133-7

[18] Kondo S, Saito A, Asada R, Kanemoto S, Imaizumi K (2011) Physiological unfolded protein response regulated by OASIS family members, transmembrane bZIP transcription factors. IUBMB life 63: 233–239. 21438114 10.1002/iub.433

[19] Asada R, Kanemoto S, Kondo S, Saito A, Imaizumi K (2011) The signaling from endoplasmic reticulum-resident bZIP transcription factors involved in diverse cellular physiology. J Biochem 149: 507–518. 21454302 10.1093/jb/mvr041

[20] Kondo S, Murakami T, Tatsumi K, Ogata M, Kanemoto S, et al (2005) OASIS, a CREB/ATF-family member, modulates UPR signaling in astrocytes. Nat Cell Biol 7: 186–194. 15665855 10.1038/ncb1213

[21] Murakami T, Kondo S, Ogata M, Kanemoto S, Saito A, et al (2006) Cleavage of the membrane-bound transcription factor OASIS in response to endoplasmic reticulum stress. J Neurochem 96: 1090–1100. 16417584 10.1111/j.1471-4159.2005.03596.x

[22] Saito A, Hino S, Murakami T, Kondo S, Imaizumi K (2007) A novel ER stress transducer, OASIS, expressed in astrocytes. Antioxid Redox Signal 9: 563–571. 17330990 10.1089/ars.2006.1520

[23] Honma Y, Kanazawa K, Mori T, Tanno Y, Tojo M, et al (1999) Identification of a novel gene, OASIS, which encodes for a putative CREB/ATF family transcription factor in the long-term cultured astrocytes and gliotic tissue. Brain Res Mol Brain Res 69: 93–103. 10350641 10.1016/s0169-328x(99)00102-3

[24] Nikaido T, Yokoya S, Mori T, Hagino S, Iseki K, et al (2001) Expression of the novel transcription factor OASIS, which belongs to the CREB/ATF family, in mouse embryo with special reference to bone development. Histochem Cell Biol 116: 141–148. 11685542 10.1007/s004180100279

[25] Chihara K, Saito A, Murakami T, Hino S, Aoki Y, et al (2009) Increased vulnerability of hippocampal pyramidal neurons to the toxicity of kainic acid in OASIS-deficient mice. J Neurochem 110: 956–965. 19549009 10.1111/j.1471-4159.2009.06188.x

[26] Murakami T, Saito A, Hino S, Kondo S, Kanemoto S, et al (2009) Signaling mediated by the endoplasmic reticulum stress transducer OASIS is involved in bone formation. Nat Cell Biol 11: 1205–1211. 19767743 10.1038/ncb1963

[27] Murakami T, Hino S, Nishimura R, Yoneda T, Wanaka A, et al (2011) Distinct mechanisms are responsible for osteopenia and growth retardation in OASIS-deficient mice. Bone 48: 514–523. 21047569 10.1016/j.bone.2010.10.176

[28] Funamoto T, Sekimoto T, Murakami T, Kurogi S, Imaizumi K, et al (2011) Roles of the endoplasmic reticulum stress transducer OASIS in fracture healing. Bone 49: 724–732. 21708301 10.1016/j.bone.2011.06.012

[29] Saito A, Kanemoto S, Kawasaki N, Asada R, Iwamoto H, et al (2012) Unfolded protein response, activated by OASIS family transcription factors, promotes astrocyte differentiation. Nat Commun 3: 967–979. 22828627 10.1038/ncomms1971

[30] Rutkowski DT, Hegde RS (2010) Regulation of basal cellular physiology by the homeostatic unfolded protein response. J Cell Biol 189: 783–794. 20513765 10.1083/jcb.201003138 PMC2878945

[31] Hotamisligil GS (2010) Endoplasmic reticulum stress and the inflammatory basis metabolic disease. Cell 140: 900–917. 20303879 10.1016/j.cell.2010.02.034 PMC2887297

[32] Ferrara N, gerber HP (2001) The role of vascular endothelial growth factor in angiogenesis. Acta Haematol 106: 148–156. 11815711 10.1159/000046610

[33] Hanahan D, Folkman J (1996) Patterns and emerging mechanisms of the angiogenic switch during tumorigenesis. Cell 86: 353–364. 8756718 10.1016/s0092-8674(00)80108-7

[34] Carmeliet P (2005) Angiogenesis in life, disease and medicine. Nature 438: 932–936. 16355210 10.1038/nature04478

[35] Harper SJ, Bates DO (2008) VEGF-A splicing: the key to anti-angiogenic therapeutics? Nat Rev Cancer 8: 880–887. 18923433 10.1038/nrc2505 PMC2613352

[36] Folkman J (1971) Tumor angiogenesis: therapeutic implications. N Engl J Med 285: 1182–1186. 4938153 10.1056/NEJM197111182852108

[37] Ferrara N, Davis-Smyth T (1997) The biology of vascular endothelial growth factor. Endocr Rev 18: 4–25. 9034784 10.1210/edrv.18.1.0287

[38] Ghosh R, Lipson KL, Sargent KE, Mercurio AM, Hunt JS, et al (2010) Transcriptional regulation of VEGF-A by the unfolded protein response pathway. PLoS One 5: e9575. 20221394 10.1371/journal.pone.0009575 PMC2833197

[39] Pereira ER, Liao N, Neale GA, Hendershot LM (2010) Transcriptional and post-transcriptional regulation of proangiogenic factors by the unfolded protein response. PLoS One 5: e12521. 20824063 10.1371/journal.pone.0012521 PMC2932741

[40] Newman JR, Keating AE (2003) Comprehensive identification of human bZIP interactions with coiled-coil arrays. Science 300: 2097–2101. 12805554 10.1126/science.1084648

[41] Krzystolik MG, Afshari MA, Adamis AP, Gaudreault J, Gragoudas ES, et al (2002) Prevention of experimental choroidal neovascularization with intravitreal anti-vascular endothelial growth factor antibody fragment. Arch Ophthalmol 120: 338–346. 11879138 10.1001/archopht.120.3.338

[42] Ferrara N, Damico L, Shams N, Lowman H, Kim R (2006) Development of ranibizumab, an anti-vascular endothelial growth factor antigen binding fragment, as therapy for neovascular age-related macular degeneration. Retina 26: 859–870. 17031284 10.1097/01.iae.0000242842.14624.e7

[43] Zhang X, Bao S, Lai D, Rapkins RW, Gillies MC (2008) Intravitreal triamcinolone acetonide inhibits breakdown of the blood-retinal barrier through differential regulation of VEGF-A and its receptors in early diabetic rat retinas. Diabetes 57: 1026–1033. 18174522 10.2337/db07-0982 PMC2836241

[44] Lin JH, Li H, Yasumura D, Cohen HR, Zhang C, et al (2007) IRE1 signaling affects cell fate during the unfolded protein response. Science 318: 944–949. 17991856 10.1126/science.1146361 PMC3670588

[45] Zode GS, Kuehn MH, Nishimura DY, Searby CC, Mohan K, et al (2011) Reduction of ER stress via a chemical chaperone prevents disease phenotypes in a mouse model of primary open angle glaucoma. J Clin Invest 121: 3542–3553. 21821918 10.1172/JCI58183 PMC3163970

[46] Li J, Wang JJ, Yu Q, Wang M, Zhang SX (2009) Endoplasmic reticulum stress is implicated in retinal inflammation and diabetic retinopathy. FEBS Lett 583: 1521–1527. 19364508 10.1016/j.febslet.2009.04.007 PMC2691649

[47] Zhong Y, Li J, Chen Y, Wang JJ, Ratan R, Zhang SX (2012) Activation of endoplasmic reticulum stress by hyperglycemia is essential for Müller cell-derived inflammatory cytokine production in diabetes. Diabetes 61: 492–504. 22228718 10.2337/db11-0315 PMC3266398

[48] Salminen A, Kauppinen A, Hyttinen JM, Toropainen E, Kaarniranta K (2010) Endoplasmic reticulum stress in age-related macular degeneration: trigger for neovascularization. Mol Med 16: 535–542. 20683548 10.2119/molmed.2010.00070 PMC2972399

[49] Saito A, Hino S, Murakami T, Kanemoto S, Kondo S, et al (2009) Regulation of endoplasmic reticulum stress response by a BBF2H7-mediated Sec23a pathway is essential for chondrogenesis. Nat Cell Biol 11: 1197–1204. 19767744 10.1038/ncb1962

[50] Ikeda F, Nishimura R, Matsubara T, Tanaka S, Inoue J, et al (2004) Critical roles of c-jun signaling in regulation of NFAT family and RANKL-regulated osteoclast differentiation. J Clin Invest 114: 475–484. 15314684 10.1172/JCI19657 PMC503767

