# Supplementary material for: Transcriptional Regulation of VEGFA by the Endoplasmic Reticulum Stress Transducer OASIS in ARPE-19 Cells
Source: PLoS One. 2013 Jan 30;8(1):e55155. doi: 10.1371/journal.pone.0055155 (PMC3559390; doi:10.1371/journal.pone.0055155)
Supplement: Table S1 — RT-PCR primer sequences. The following table indicates sets of primers used for RT-PCR. (DOC) [file pone.0055155.s001.doc]

**Table S1. RT-PCR Primer Sequences.**

| **Gene** | **Forward Sequence 5'-3'** | **Reverse Sequence 5'-3'** |
| --- | --- | --- |
| VEGFA | CAAGTGGTCCCAGGCTGCAC | AGCTCATCTCTCCTATGTGC |
| β-actin | TCCTCCCTGGAGAAGAGCTA | TCCTGCTTGCTGATCCACAT |
| XBP1 | CAGCGCTTGGGGATGGATGC | CCATGGGGAGATGTTCTGGA |
| ATF4 | GGACAGATTGGATGTTGGAGAAAATG | GGAGATGGCCAATTGGGTTCAC |
| ATF6 | GGATTTGATGCCTTGGGAGTCAGAC | ATTTTTTTCTTTGGAGTCAGTCCAT |
| OASIS | GAACATGGAGGACTTCTCCAATG | CGGGCTCTGCTCCTGCTTCAC |
| CREBH | GGATGAGAAGAAGCTGCTGG | AGGATGATGAGGGCAAAGGAC |
| CREB4 | CCTCACCAAGGCAGAGGAGAG | CTGGGCAGGATGATGAGAGCC |
